# Precarious work and Covid-19: repercussions on the health of primary health care workers

**DOI:** 10.11606/s1518-8787.2026060006692

**Published:** 2026-04-17

**Authors:** Ticiane Teixeira de Mendonça, Sandra Garrido de Barros, Thais Regis Aranha Rossi, Ana Clara de Rebouças de Carvalho, Denise Nogueira Cruz, Laio Magno, Ines Dourado

**Affiliations:** I Universidade do Estado da Bahia. Departamento de Ciências da Vida. Salvador, BA, Brasil; II Secretaria Municipal de Saúde de Salvador. Salvador, BA, Brasil; III Universidade Federal da Bahia. Faculdade de Odontologia. Departamento de Odontologia Social e Pediátrica. Salvador, BA, Brasil; IV Universidade Federal da Bahia. Instituto de Saúde Coletiva. Salvador, BA, Brasil; V Fundação Oswaldo Cruz. Instituto Gonçalo Muniz. Salvador, BA, Brasil

**Keywords:** COVID-19, Occupational Health, Health Personnel, Primary Health Care

## Abstract

**OBJECTIVE:**

To analyze how the Covid-19 pandemic influenced the working conditions and health of primary health care professionals in a health district of a capital city in Northeast Brazil.

**METHODS:**

This was a qualitative study using the theoretical framework of the health work process, part of the formative research of the project "Expansion of testing, quarantine, e-health and telemonitoring strategies in the fight against Covid-19 in Brazil" (TQT Project). Data were collected between December 2021 and March 2022 in a health district of a capital city in Northeast Brazil to support the implementation of the aforementioned project. Twenty-two interviews and three focus groups with 19 participants, all health care professionals from the health district under study, were analyzed for their content.

**RESULTS:**

The pandemic context caused repercussions on working conditions and health, with the effects of precarious working conditions and the implementation of protective measures being highlighted as key elements in the dimension of working conditions. Mental suffering, the impact of gender issues, and post-Covid-19 conditions were identified as the main elements contributing to the illness of the professionals.

**CONCLUSION:**

The evidence set gathered in this study indicates that various stressors emerge or worsen intensely and inseparably among health care professionals, clearly demonstrating the relationship between the pandemic and the health-disease process of these workers. Therefore, it is imperative to reverse the situation of illness among workers in the post-pandemic period to mitigate its effects in the medium and long term.

## INTRODUCTION

The Covid-19 pandemic prompted an unprecedented health, political, and social reorganization. The health crisis amplified existing difficulties in health services, especially in countries with public systems weakened by decades of underfunding and exposed those responsible for directly confronting it on a daily basis: health care workers.

Previous contexts of health crises help to gauge the short- and long-term impact of the repercussions of this pandemic on workers’ health^1^. The precariousness of work became prominent among these consequences, intensifying or worsening during the pandemic context, in all its dimensions^1,2^.

It is important to contextualize the chronological sequence of the health emergency in Brazil. The evolution of the pandemic presented three peaks of deaths (February to July/2020, November/2020 to April/2021, and December/2021 to May/2022), with the second wave being the most lethal of all. The introduction of the vaccine, at the beginning of 2021, influenced the reduction in the mortality rate and consequently in the demand for hospital services, increasing the demand for primary and secondary care levels^3^.

The health care workforce in Brazil, in addition to the labor aspects involving the exposure of professionals, faces a deterioration of working conditions: precarious employment, irregular and extended working hours, and temporary contracts, among other factors^4^. The Brazilian political, economic, and labor contexts amplified the vulnerability of professionals, since the 2017 labor reform institutionalized employment relationships with reduced or no social protection and workers’ rights^5^.

It is also discussed, in the context of the pandemic, that the stress resulting from work overload and the absence of protocols made the teams more susceptible to psychological distress and increased anxiety. This dynamic may have increased the risks of mental illness as well as aggravated existing problems. Additional stressors, such as fear of infection and limitations in the support network, may have implied greater physical and emotional exposure to the new virus^6^.

This study aimed to analyze how the Covid-19 pandemic influenced the working conditions and health of primary health care (PHC) professionals in a health district of a capital city in Northeast Brazil.

## METHODS

This was a qualitative study of the working conditions and health of PHC professionals in a health district of a capital city in Northeast Brazil. This article presents the results of the formative research, which aimed to support the definition of the best strategies to implement the intervention "Expansion of testing, quarantine, e-health and telemonitoring strategies in the fight against Covid-19 in Brazil" (TQT Project).

The formative research stage that gave rise to this study was conducted between December 2021 and March 2022 and consisted of semi-structured interviews with professionals from the 22 basic health units (BHU) of the health district and three focus groups (FGs) with workers from those units who had performed Covid-19 testing at some point. Participants were indicated by the unit managers, with eligibility criteria including at least one year of work at the unit and participation in activities related to the fight against Covid-19. Interviews and FGs were conducted in person, maintaining all health safety measures (use of personal protective equipment, hygiene, and social distancing), considering the context of the health crisis.

The semi-structured interview script included personal and functional identification, aspects of the work process, conceptions and practices in facing the pandemic, and measures adopted by the Municipal Health Department to protect professionals working on the front lines in the fight against Covid-19.

For the FGs, the script aimed to delve deeper into the strategies used in the units where testing was carried out, in an attempt to identify, among other elements, changes in the work process. It is noteworthy that, in the municipality, testing did not occur in all BHU. For each FG, at least two health professionals and the manager of two units where testing was performed were invited. These meetings took place in the health services or at a public university located in the same health district.

The interviews and FGs were recorded, transcribed, reviewed, and coded to avoid identifying the participants. The BHU were numbered from 1 to 22, while the FGs were identified from 1 to 3. Subsequently, the research corpus was subjected to content analysis^7^.

Considering that facing Covid-19 implied new arrangements within health units, amplifying the precariousness of health work (inadequate working conditions, salary, and safety), with a lack of protective equipment, supplies, beds, infrastructure, and personnel, as well as extensive working hours^8^, and their relationship with the health-disease process, especially in mental illness^9^, the following analytical categories were defined: working conditions, addressing precariousness and institutional measures for worker protection and health conditions, including mental health, gender implications in the health-disease process, and long Covid, as detailed in Chart 1.

**Chart 1:** Categories for analyzing the working conditions and health of health care professionals in a health district of a capital city in Northeast Brazil, 2020–2022.

Dimensions	Subdimensions	Evidence	Example
Working conditions	Precarious employment	Precariousness in labor relations before or during the pandemic	"Furthermore, here, I'm an LP, so I don't have the same protection as a public servant. I have all the obligations of a public servant, but I don't have the advantages of a public servant." (BHU 17, female, doctor)
	Institutional measures adopted for the protection of workers	Institutional protective measures offered when confronting the health crisis	"They created a working group at the Secretariat to provide emotional support and work with the teams that felt emotionally vulnerable during this Covid-19 routine." (BHU 19, female, social worker)
Health conditions	Mental health	Repercussions on mental health	"Oh, another thing I forgot to mention: many people were depressed during the pandemic, many people with psychiatric disorders." (BHU 17, female, doctor)
	Gender implications in the health-disease process	Relationship between gender and health conditions	"With the whole process happening inside their homes, these are children who needed someone at home, because they were having online classes, I went through that myself, right?" (BHU 10, female, nurse)
	Long Covid	Sequelae after Covid-19 infection	"Many of our colleagues have serious after-effects, and I don't even know if they'll be able to return to work." (BHU 13, female, social worker)

LP: legal person; BHU: basic health unit.

Accordingly, the content analysis sought to highlight the relationships between precarious working conditions and workers’ illness, as proposed by Franco et al.^9^, as well as the worsening of these conditions in the face of the health crisis and the changes in labor relations driven by the 2017 labor reform, as analyzed by Druck^8^. On the other hand, the study aimed to explain the institutional measures for worker protection adopted during the Covid-19 pandemic.

The research project was approved by the Research Ethics Committee of the *Instituto de Saúde Coletiva da Universidade Federal da Bahia*, under No. 53844121.4.1001.5030. All participants signed an informed consent form.

## RESULTS

The study revealed inadequate working and safety conditions and lack of personal protective equipment, supplies, infrastructure, and personnel, as well as long working hours. It also identified the hiring of professionals under precarious employment conditions, which led to illness among the PHC workforce. Professionals pointed to the insufficiency of worker protection measures, whether due to differentiated treatment of permanent and contract employees compared to professionals hired during the pandemic (those working as independent contractors), or due to the suspension of services established after the most critical period of the pandemic.

### Characterization of Participants

This study included 39 health care professionals from 22 BHU in the intervention health district: 20 participated only in interviews; 17 participated in focus groups (FGs); and two participated in both interviews and FGs. The majority of participants were Black women (Black and/or mixed-race). Regarding their roles, the highest participation in the interviews was from nurses, dentists, and doctors, while for the focus groups, it was from nurses, managers, and dentists. The participants’ ages ranged from 28 to 62 years (Table).

**Table:** Profile of study participants. Salvador, Bahia, Brazil, 2022.

Variables		Professional interviews (22 participants)		Focus groups (19 participants)	
		n	%	n	%
Sex					
	Female	21	95.5	15	78.9
	Male	1	4.5	4	21.1
Skin color					
	Black	10	45.5	2	10.5
	Brown	6	27.3	14	73.7
	White	4	18.2	2	10.5
	No information	2	9.0	-	-
	Indigenous	-	-	1	5.3
	Yellow	-	-	-	-
Job title					
	Nurse	12	54.5	7	36.8
	Dentist	4	18.2	4	21.1
	Docter	3	13.6	1	5.3
	Social worker	2	9.1	1	5.3
	Nursing technician	1	4.5	1	5.3
	Manager	-	-	5	26.2

### Working Conditions

The harmful effects on workers’ health resulting from precarious work were highlighted in the professionals’ statements. The insufficiency of human resources exposes workers to long and exhausting working hours, a situation aggravated in the pandemic scenario, when vacation time was suspended and/or inadequate rest periods were granted.

There is no vacation replacement […] There is no retirement replacement. Right? There is no leave of absence replacement. […] we went two years without being able to do that. So, the HR deficit is very large […] It ends up overloading other people (BHU 21, female, nurse)

Precarious forms of employment, such as hiring workers as independent contractors, resulted in a lack of labor protections for professionals, including the right to health care. These professionals, who were there to promote health, were financially penalized for taking time off work to undergo diagnostic tests or receive medical care.

Here, I am an legal person […]. I have all the obligations of a public servant, but I don't have the advantages of a public servant. […] If I get sick with Covid, I can take time off and they pay for it. But if I get sick with anything else, […] I lose the days I miss. Those who care for others are not cared for themselves. […] Now they are providing support for those who are public employees (BHU 17, female, doctor)

The workers’ accounts highlight the creation of a specific institutional framework for protecting workers’ health during the Covid-19 pandemic, although this was implemented late and was insufficient.

This whole issue of putting the professional on the front lines, like that, was a big exposure […] I think almost a year later […] With NAAT, this made things much easier, because you arrive at the scheduled time, you go in and you get evaluated (BHU 16, female, nurse).

Several employees were becoming emotionally ill, with a very high level of burnout […]. They formed a working group within the Secretariat to provide emotional support and work with teams that felt emotionally vulnerable during this Covid-19 routine (BHU 19, female, social worker).

Regarding this specific service (Support and Assistance Center for Workers — NAAT), established during the pandemic to protect health care workers, there is a convergence of opinions regarding its importance in providing care. The interviewees also highlighted the importance of continuing to offer this service after the pandemic, aiming to ensure the continued health care of health care professionals.

It's a good thing it existed, but it still needed to exist in a stronger way […]. It's an essential thing, it can't end, it will have to be forever (BHU 03, female, doctor).

It was quite important in terms of providing support […], these guidelines. It was the only place where we had this contact with management, because the NAAT represented management for us, right? […] I think it was very important that it existed. There were strategies for testing professionals, which I think was an important support system that the municipality created (BHU 04, female, nurse).

### Health Conditions

During the pandemic, health care professionals were frequently exposed to exhausting work and a context of high stress, requiring the incorporation of new knowledge and skills (Chart 2).

**Chart 2:** Dimensions related to the health conditions of health care professionals in a health district in a capital city of Northeast Brazil, 2020–2022.

Dimension	Subdimension	Evidence related to workers’ health
Health conditions	Mental health	**2.1 — Work process and its repercussions on the health of professionals** "Ultimately, I think the first impact was this issue of resizing activities, of relearning the work. Not reprimanding, no. Learning in the face of this new reality, which was a pandemic reality. The second point, for the health teams, was having the stamina to cope with the service without a prospect of it ending […]." (BHU 10, female, social worker) "[…] what I found very difficult was the stress generated in health care professionals, right? And because of the insecurity, the lack of knowledge of how to deal with the situation, right? Because of the workload, and there was a heavy workload […]." (BHU 9, female, nurse) **2.2 — Challenges faced by health care workers in the context of the pandemic** "So, I was afraid that we would get sick and they would transmit it, that my parents would catch it [….]." (BHU 2, female, nurse) "Without doing physical activity, stuck at home, only going out to work and coming back […]. So, I think that was the most difficult part […]." (BHU 5, female, nurse) "In reality, for me, Covid-19, the disease, was a big shock. It was unexpected, it was frightening, challenging, because, besides being on the front lines in everything, we, in addition to being professionals dealing with this new disease, had all the fears that ordinary people have […]." (BHU 1, female, dentist) "In my life, in particular, it was devastating, because I lost my mother to Covid. And it was at the time I was working. […] we were forced to work without any support, without any training, without basic materials to work with […]." (BHU 21, female, nurse) **2.3 — Physical and emotional exhaustion of the health care workforce** "And another thing that is making the nursing team very tired, in particular, is the issue of vaccination, because the same vaccination team is the same team that is providing care here, it's the same team that works on weekends. We vaccinated against Covid here for eight months, from January to August, so we were exhausted […]." (BHU 2, female, nurse) "I think it was the most challenging of all, because working relationships became extremely strained and tense. Everyone felt extremely overwhelmed, tired, and unfairly treated […]." (BHU 16, female, nurse) **2.4 — Impacts on mental health** "We were going to have to do testing. So, it was really desperate…A feeling of fear, of panic. Several colleagues suffered from depression. We had colleagues who were on leave due to depression, right? I had a panic attack at first, but I didn't develop depression, I didn't have any mental health problems, but I was very desperate… Everyone had that moment of panic. And, you know, when they talk about heroes, they don't talk about the heroes who are in the basic health unit, do they? People value those who are in hospitals, but they need to understand that we are also exposed […]." (BHU 12, female, dentist) "Burnout syndrome is prevalent, and there are several cases of colleagues who are ill and absent from work, right? As a result of the pandemic in general and everything it generates. But this in itself has caused an overload on those who remain, and who are falling ill with other health problems, right? And, therefore, this lack of recognition of the professionals who are there giving their all, right? It's very disappointing! Because the professionals are working hard and are there on the front lines, truly on the front lines. But this lack of recognition…So, the overload is on Saturdays, on Sundays, and you have to work no matter what and you have to deal with it. And we can't, you know? At some point… Really… Mental health, right? […]." (FG 02, BHU 18, female, nurse)
	Gender implications in the health-disease process	**2.5 — Influence of social gender markers on the health of female workers** "[…] I won't be able to manage, because I'm working; you have no idea of the pace I'm working at there, without any days off, without anything; how am I going to keep up with the girls in this technology matter? […]." (BHU 16, female, nurse) "I think, in my experience, I had to maintain a great balance… With all the processes within your home, there were children who needed someone at home because they were having online classes; I went through that, right? […]." (BHU 10, female, nurse)
Health conditions	Long Covid	**2.6 — Long Covid** "I have shortness of breath, which I still have today. I climbed these stairs here, which are ten steps. When I reach the last step, I have to stop to regulate my breathing before I can continue. I had severe tachycardia; the other day I was here, and my heart rate was 180 beats per minute. And memory loss. I went to a neurologist and everything, but I was left with some after-effects. There's no follow-up care, as the neurologist told me. They don't have medication to deal with it, there's no treatment to do, because even today we have to learn to live with it, right? And since we're in the health care field, we can understand this […]." (BHU 21, female, nurse) "I'm telling you, after you have Covid, you're never the same person again. I'm saying this, I'm saying that my hair fell out… Yes, my hair fell out. I have a colleague who said she became forgetful. So, it's about expanding this psychological support for the employees, even for the patients […]. I know people who say they developed insomnia […]." (BHU 18, female, nursing technician) "Many of our colleagues have serious after-effects, and I don't even know if they'll ever be able to return to work […]." (BHU 13, female, social worker)

BHU: basic health unit; FG: focus group.

In addition to changes in daily routines and work processes, PHC professionals who worked on the front lines in the fight against Covid-19 had concerns related to the fear of becoming infected and infecting others, leading to deprivation of family contact, constant changes in care protocols due to the lack of knowledge about the disease, and a lack of personal protective equipment. This scenario of extreme pressure was responsible for intense emotional exhaustion among workers and persisted for much of the pandemic, but especially in the period before vaccination (Chart 2, 2.2).

With the progression of the pandemic, the health care workforce was subjected to chronic stress, and the exhaustion caused by prolonged working hours impacted work relationships and emotional well-being, making PHC workers even more vulnerable. The suspension of vacation and leave periods for much of the pandemic further exacerbated fatigue and illness among workers (Chart 2, 2.3).

It was observed that fear, panic, and their repercussions were part of the narrative of the professionals who worked in PHC. Psychological changes appeared prominently, especially depression and feelings of worthlessness. The pandemic made health care environments more overloaded, which had a reciprocal relationship with the illness of the workers. (Chart 2, 2.4).

Inequality was observed in the manifestation of illness among male and female workers. The reports highlighted the accumulation of responsibilities for female workers also in the domestic sphere, making the consequences of the pandemic even more severe among women, particularly while measures to suspend collective activities such as schools, daycare centers, and other social services remained in place. (Chart 2, 2.5).

In addition to physical and emotional stress resulting from the work environment during the pandemic, some workers who had Covid-19 have continued to experience prolonged symptoms, presenting a picture of long Covid. The interviews highlighted symptoms such as shortness of breath, memory loss, and insomnia, among other health conditions. In this context, some professionals reported difficulty in carrying out their work activities (Chart 2, 2.6).

## DISCUSSION

The main findings of this study demonstrated that the worsening of precarious working conditions in health care and the insufficiency of institutional measures to protect professionals during the health emergency had repercussions on their mental health, especially among women.

There is no doubt that the pandemic made the vulnerabilities related to working conditions and health even more evident. Precarious work has its own dimensions and can be understood as a reflection of productive restructuring^2^. Exhaustion derived from prolonged working hours, exacerbated by social unprotectedness and emotional problems, negatively impacted the health of workers, generating impacts that went beyond the biological aspect of the health-disease process^9^.

Employment contracts through legal entities or temporary contracts contributed to increasing the vulnerability of professionals, compromising their remuneration after absences due to illness, including Covid-19 testing^10^. In general, the employment of health care workers through these types of contracts is accompanied by a discourse of flexibility and freedom, often appreciated by workers, although, in practice, they promote work overload, transfer of risks and costs to the workers themselves because of the suppression of labor rights^11^.

These fragile and unprotected employment relationships lead to presenteeism, which consists of the act of the professional attending work with signs and/or symptoms of illness, a phenomenon that occurs in various places and negatively impacts the health of the worker and the institutions^12^. The absence of social protection and the fear of losing their jobs cause individuals, including doctors as pointed out in this study, to attend work even when their absence is necessary or recommended, as indicated in the statement of one of the interviewed professionals (Chart 2)^13^.

It is also important to add the health repercussions derived from precarious employment, since social unprotectedness can compromise access to health care and remuneration during periods of absence from work.

Another important point in addressing the pandemic is the physical and emotional protection of workers. During the health crisis, frontline professionals experienced an overload of negative emotions in their work environments, which demanded psychological care from the beginning of the problem^14^. Institutional measures to protect workers are essential for overcoming a pandemic.

In line with the federal context^15^, the Municipal Health Secretariat published a Contingency Plan for Workers in 2020, with one of its main objectives being to preserve the workforce of the municipal network, including the physical and mental health of employees. A service was then created, perceived by the workers as a space offering testing, psychological and psychiatric support during the health crisis, which operated between May 2020 and November 2021, when it was discontinued^16^.

In December 2023, the municipality published a technical note with general guidelines on Covid-19 prevention and control measures, but it did not include the protection of health care professionals in the prevention and control measures. In this document, the service providing exclusive care to Municipal Health Secretariat workers appeared as an offer of testing for professionals, demonstrating an institutional setback in the care of workers.

The lack of commitment from managers to the proposed interventions constitutes a difficulty in the continuity of worker protection actions^17^. This shows that, despite the legal framework available for the execution of the activities foreseen in the National Policy on Workers’ Health^18^, the practice of protecting professionals is permeated by difficulties.

The illness of health care professionals can reduce the human resources available to confront the pandemic and compromise the response of health services. The new work context resulting from the pandemic imposed the need to learn new care protocols, given the lack of knowledge about the disease. Individuals who formed the health care workforce lived with fear, anxiety, and work overload, among other factors that can be considered triggers for the onset or worsening of mental illnesses^19,20^. Protecting the physical and mental health of health care workers is of fundamental importance for maintaining services and preserving human life, especially in contexts of health crises.

Health care professionals also faced a scarcity of personal protective equipment, social isolation, and extensive working hours^21^. Furthermore, the narratives highlighted the workload during much of the health crisis. The extension of working hours reduced the possibility of maintaining a healthy lifestyle, increasing the risk of illness^22^. The International Labour Organization^23^ emphasizes that individuals who work 48 hours a week, whether in person or virtually, suffer negative consequences in the short and long term.

The increase in stress levels in the work environment results in three main problems: mental exhaustion, anxiety disorders, and depression^21^. The prevalence of symptoms of mental disorders, such as anxiety and depression, in PHC professionals during the pandemic was around 43.2%, and workload is one of the elements associated with the presence of these disorders^24^.

A review article on the mental health of frontline workers showed prevalence of anxiety symptoms ranging from 44.6 to 62%, with about 30% of professionals presenting moderate or severe levels of anxiety, and 50% presenting symptoms of depression^25^.

In this context, the World Health Organization^26^ indicated a global increase in the prevalence of anxiety and depression of 25% in the first year of the pandemic. Adherence to physical distancing measures led to interruptions in mental health services, creating gaps in the treatment offered to the population. It is essential to expand mental and psychosocial health services, recognizing them as components of universal health coverage worldwide.

Chronic stress from work led some workers to develop burnout syndrome, or professional exhaustion syndrome. In general, the impact of mental illness on health care professionals has social, personal, and institutional consequences, affecting the well-being of the people they serve^27^.

Therefore, it is observed that mental health should be considered one of the central elements in public policies for worker health within the Unified Health System, given the essential nature of the service these professionals provide in caring for the population. Considering the health conditions of the workers, the influence of gender on the illness of female workers is evident.

The findings of this study corroborate the reviewed literature^22,28,29^ on the participation of women in the health sector: they are the majority. In the context of the health crisis, the accumulation of work responsibilities, low wages, lack of sharing of domestic tasks, among other elements, contributed to increasing the risk of infection and repercussions on the physical and mental health of female workers^28^.

Considering the various types of violence to which they are exposed daily, women are three times more likely to experience minor mental health disorders compared to men^30^. Therefore, the analysis of the pandemic's impact on workers’ health through a gender lens exposes inequities and vulnerabilities that permeate work practices and life in society.

The identification of workers with prolonged Covid-19 symptoms is also highlighted. In Brazil, the Ministry of Health opted for the term "post-Covid conditions" to describe health changes that persist or begin four weeks or more after Covid-19 infection, and that cannot be classified under another diagnosis^31^. Considering the importance of this condition in the work context, the European Agency for Safety and Health at Work developed guidelines for managers and employers on the safe and healthy return of professionals with persistent symptoms to the work environment, including maintaining contact with the worker during their absence and preparing for their return, considering strategies such as phased (or gradual) return and adjustments to functions and schedules^32^.

This condition also has implications for the development of new chronic diseases, and consequently, health services must be prepared to deal with these new demands^33^, as well as the companies and services where these professionals are employed.

As for limitations of this study, it should be noted that the professionals interviewed belonged to a single health district, and that expanding the sample could generate more robust reflections on the dimensions highlighted. However, the evidence of the potential health impairment of workers infected with Covid-19 indicates the need for monitoring these professionals and establishing coordinated care actions in the post-Covid context.

Analyzing the health and working conditions of PHC professionals, it was observed that the unique context of the pandemic amplified illness and made room for new stressors. It is worth noting that the precariousness of work, and especially of health care work, is the result of a historical process and not restricted to Brazil. Understanding the implications of the advancement of neoliberal policies and their impact on workers’ health and their work process is essential for improving public policies and the quality of care provided. The results of this research contribute to understanding the elements that act, in an intertwined way, on workers’ health.

Although the problems described are not new, there is no doubt that the pandemic aggravated these vulnerabilities. At the center of the health crisis remained health care workers, experiencing in their daily lives problems that already affected them even before the outbreak of the pandemic.

## Data Availability

The research data not included in the article are not available.
