# Supplementary material for: Precarious work and Covid-19: repercussions on the health of primary health care workers
Source: Rev Saude Publica. 2026 Apr 17;60(Suppl 1):e11s. doi: 10.11606/s1518-8787.2026060006692 (PMC13089370; doi:10.11606/s1518-8787.2026060006692)
Supplement: Translation

# Translation: Precarização do trabalho e Covid-19: repercussões sobre a saúde dos trabalhadores de APS

**Authors:** Ticiane Teixeira de Mendonça, Sandra Garrido de Barros, Thais Regis Aranha Rossi, Ana Clara de Rebouças de Carvalho, Denise Nogueira Cruz, Laio Magno, Ines Dourado

**Affiliations:** 1 Universidade do Estado da Bahia. Departamento de Ciências da Vida. Salvador, BA, Brasil; 2 Secretaria Municipal de Saúde de Salvador. Salvador, BA, Brasil; 3 Universidade Federal da Bahia. Faculdade de Odontologia. Departamento de Odontologia Social e Pediátrica. Salvador, BA, Brasil; 4 Universidade Federal da Bahia. Instituto de Saúde Coletiva. Salvador, BA, Brasil; 5 Fundação Oswaldo Cruz. Instituto Gonçalo Muniz. Salvador, BA, Brasil

**Keywords:** COVID-19, Saúde Ocupacional, Pessoal de Saúde, Atenção Primária à Saúde

## Abstract

**OBJETIVO:**

Analisar como a pandemia de Covid-19 influenciou as condições de trabalho e saúde de profissionais da atenção primária à saúde de um distrito sanitário de uma capital do Nordeste.

**MÉTODOS:**

Trata-se de um estudo de abordagem qualitativa, utilizando o referencial teórico do processo de trabalho em saúde, parte da pesquisa formativa do projeto "Expansão das estratégias de testagem, quarentena, *e-health* e telemonitoramento no enfrentamento da Covid-19 no Brasil" (Projeto TQT). Os dados foram coletados entre dezembro/2021 e março/2022 em um distrito sanitário de uma capital do Nordeste do Brasil para subsidiar a implementação do referido projeto. Foram analisadas em seu conteúdo 22 entrevistas e três grupos focais com 19 participantes, todos profissionais de saúde do distrito sanitário em estudo.

**RESULTADOS:**

O contexto da pandemia causou repercussões nas condições de trabalho e saúde, sendo observados, na dimensão condições de trabalho, os efeitos da precarização e da instituição de medidas protetivas como elementos de destaque. O sofrimento mental, o impacto das questões de gênero e as condições pós-Covid-19 foram identificados como principais elementos do adoecimento dos profissionais.

**CONCLUSÃO:**

O conjunto de evidências reunidas neste estudo aponta que diversos estressores surgem ou se agravam de forma intensa e indissociável entre os profissionais de saúde, ficando clara a relação da pandemia com o processo saúde-doença dos trabalhadores. Torna-se, portanto, imperioso reverter o quadro de adoecimento dos trabalhadores no pós-pandemia, de modo a mitigar seus efeitos em médio e longo prazos.

## INTRODUÇÃO

A pandemia de Covid-19 promoveu uma reorganização sanitária, política e social sem precedentes. A crise sanitária ampliou as dificuldades já existentes nos serviços de saúde, sobretudo em países com sistemas públicos enfraquecidos por décadas de subfinanciamento, além de expor os responsáveis pelo enfrentamento direto do seu cotidiano: os trabalhadores de saúde.

Contextos anteriores de crise sanitária ajudam a dimensionar o impacto, em curto e longo prazos, das repercussões dessa pandemia na saúde dos trabalhadores^1^. A precarização do trabalho assumiu eminência no conjunto dessas consequências, acentuando-se ou agravando-se durante o contexto pandêmico, em todas as suas dimensões^1,2^.

É importante contextualizar a sequência cronológica da emergência sanitária no Brasil. A evolução da pandemia apresentou três picos de óbitos (fevereiro a julho/2020, novembro/2020 a abril/2021 e dezembro/2021 a maio/2022), sendo a segunda onda a mais letal de todas. A introdução da vacina, no início de 2021, influenciou a redução da taxa de mortalidade e consequentemente de atendimento em serviços hospitalares, aumentando a demanda para os níveis de atenção primária e secundária^3^.

A força de trabalho em saúde no Brasil, além dos aspectos laborais que envolvem a exposição dos profissionais, enfrenta deterioração das condições de trabalho: precariedade do emprego, irregularidade e extensão da carga horária e contratos temporários, entre outros fatores^4^. As conjunturas política, econômica e trabalhista brasileira potencializaram a vulnerabilidade dos profissionais, uma vez que a reforma trabalhista de 2017 institucionalizou vínculos com redução ou sem proteção social e direitos dos trabalhadores^5^.

Discute-se ainda, no contexto pandêmico, que o estresse decorrente da sobrecarga de trabalho e da ausência de protocolos tornou as equipes mais susceptíveis ao sofrimento psíquico e ao aumento da ansiedade. Essa dinâmica pode ter incrementado os riscos de adoecimento mental, bem como agravado problemas já existentes. Estressores adicionais, como medo de infectar-se e limitação da rede de apoio, podem ter implicado maior exposição física e emocional ao novo vírus^6^.

Este trabalho teve por objetivo analisar como a pandemia de Covid-19 influenciou as condições de trabalho e saúde dos profissionais de saúde da atenção primária à saúde (APS) de um distrito sanitário (DS) de uma capital do Nordeste.

## MÉTODOS

Trata-se de um estudo de abordagem qualitativa das condições de trabalho e saúde dos profissionais da APS de um DS de uma capital do Nordeste do Brasil. Este artigo apresenta resultados da pesquisa formativa, que buscou subsidiar a definição das melhores estratégias para implementar a intervenção "Expansão das estratégias de testagem, quarentena, *e-health* e telemonitoramento no enfrentamento da Covid-19 no Brasil" (Projeto TQT).

A etapa da pesquisa formativa que deu origem a este estudo consistiu, entre dezembro de 2021 e março de 2022, de entrevistas semiestruturadas com profissionais das 22 unidades básicas de saúde (UBS) do DS e três grupos focais (GFs) com trabalhadores daquelas unidades que fizeram testagem de Covid-19 em algum momento. Os participantes foram indicados pelos gerentes das unidades, tendo como critérios de elegibilidade possuir pelo menos um ano de trabalho na unidade e participado de atividades vinculadas ao enfrentamento da Covid-19. Entrevistas e GFs foram realizados presencialmente, sendo mantidas todas as medidas sanitárias de segurança (uso de equipamento de proteção individual, higiene e distanciamento social), considerando o contexto de crise sanitária.

O roteiro das entrevistas semiestruturadas incluiu identificação pessoal e funcional, aspectos do processo de trabalho, concepções e práticas no enfrentamento da pandemia e medidas adotadas pela Secretaria Municipal de Saúde para proteger os profissionais que atuavam na linha de frente no combate da Covid-19.

Para os GFs, o roteiro buscou aprofundar as estratégias usadas nas unidades onde se realizou a testagem, na tentativa de identificar, entre outros elementos, alterações do processo de trabalho. Destaca-se que, no município, a testagem não ocorreu em todas as UBS. Para cada GF, foram convidados pelo menos dois profissionais de saúde e o gerente de duas unidades em que realizaram testagens. Esses encontros ocorreram nos serviços de saúde ou em uma universidade pública localizada no próprio DS.

As entrevistas e os GFs foram gravados, transcritos, revisados e codificados de modo a evitar a identificação dos participantes. As UBS foram numeradas de 1 a 22, enquanto os GFs foram identificados de 1 a 3. Posteriormente, o *corpus* da pesquisa foi submetido à análise de conteúdo^7^.

Considerando que o enfrentamento da Covid-19 implicou novos arranjos no interior das unidades de saúde, amplificando a precarização do trabalho em saúde (condições inadequadas de trabalho, salário e segurança), com falta de equipamentos de proteção, insumos, leitos, infraestrutura e pessoal, bem como extensas jornadas de trabalho^8^, e suas relações com o processo saúde-doença, em especial no adoecimento mental^9^, foram definidas as seguintes categorias analíticas: condições de trabalho, abordando a precarização e as medidas institucionais para proteção dos trabalhadores; e condições de saúde, incluindo saúde mental, implicações de gênero no processo saúde-doença e Covid-longa, conforme detalhado no Quadro 1.

**Quadro 1:** Categorias de análise das condições de trabalho e saúde dos profissionais de saúde de um distrito sanitário de uma capital do Nordeste do Brasil, 2020–2022.

Dimensões	Subdimensões	Evidências	Exemplo
Condições de trabalho	Precarização	Precarização nas relações de trabalho anteriores ou no curso da pandemia	"Inclusive, aqui, eu sou PJ, então eu não tenho uma proteção que nem um servidor público. Eu tenho todas as obrigações de servidor público, mas não tenho as vantagens do servidor público." (UBS 17, sexo feminino, médica)
	Medidas institucionais adotadas para proteção dos trabalhadores	Medidas protetivas institucionais ofertadas durante o enfrentamento da crise sanitária	"Eles fizeram um grupo de trabalho na Secretaria para dar suporte emocional e trabalhar as equipes que se sentissem fragilizadas emocionalmente nessa rotina do Covid." (UBS 19, sexo feminino, assistente social)
Condições de saúde	Saúde mental	Repercussões em saúde mental	"Ah, outra coisa que eu esqueci de pontuar: muita gente depressiva durante a pandemia, muita gente com transtornos psiquiátricos." (UBS 17, sexo feminino, médica)
	Implicações de gênero no processo saúde-doença	Relação entre gênero e condições de saúde	"Com todo o processo dentro da sua casa, são filhos que precisavam ter alguém dentro de casa, porque estava tendo aula *online*, eu passei por isso, né?" (UBS 10, sexo feminino, enfermeira)
	Covid longa	Sequelas depois da infecção por Covid-19	"Muitos dos nossos colegas estão com sequelas graves, que eu não sei nem se volta mais *pra* trabalhar." (UBS 13, sexo feminino, assistente social)

PJ: pessoa jurídica; UBS: unidade básica de saúde.

Nesse sentido, a análise de conteúdo buscou evidenciar as relações entre condições precárias de trabalho e o adoecimento dos trabalhadores, conforme proposto por Franco et al.^9^, bem como o agravamento dessas condições diante da crise sanitária e das mudanças nas relações de trabalho alavancadas pela reforma trabalhista de 2017, como analisado por Druck^8^. Por outro lado, buscou-se explicitar as medidas institucionais de proteção do trabalhador adotadas durante a pandemia de Covid-19.

O projeto de pesquisa foi aprovado pelo Comitê de Ética em Pesquisa do Instituto de Saúde Coletiva da Universidade Federal da Bahia, sob o número 53844121.4.1001.5030. Todos os participantes assinaram o termo de consentimento livre e esclarecido.

## RESULTADOS

O estudo evidenciou condições inadequadas de trabalho e segurança, falta de equipamentos de proteção individual, insumos, infraestrutura e de pessoal, bem como extensas jornadas de trabalho, além de ter sido identificada a contratação de profissionais em regime precarizado, que implicaram em adoecimento da força de trabalho da APS. Os profissionais apontaram a insuficiência das medidas de proteção do trabalhador, seja pela conduta diferenciada de estatutários e celetistas em relação aos profissionais contratados durante a pandemia (pejotizados/ pessoas jurídicas), seja pela suspensão de serviços estabelecidos após o período mais crítico da pandemia.

### Caracterização dos Participantes

Participaram deste estudo 39 profissionais de saúde de 22 unidades de APS do DS da intervenção: 20 participaram apenas de entrevistas; 17 participaram de GFs e dois participaram tanto de entrevistas como de GFs. A maioria dos participantes eram mulheres negras (pretas e/ou pardas). Quanto ao cargo, a maior participação foi de enfermeiras, dentistas e médicos para as entrevistas, e enfermeiras, gerentes e dentistas para os GFs. A idade dos participantes variou de 28 a 62 anos (Tabela).

**Tabela:** Perfil dos participantes do estudo. Salvador, Bahia, Brasil, 2022.

Variáveis		Entrevistas profissionais (22 participantes)		Grupos focais (19 participantes)	
		n	%	n	%
Sexo					
	Feminino	21	95,5	15	78,9
	Masculino	1	4,5	4	21,1
Cor da pele					
	Preta	10	45,5	2	10,5
	Parda	6	27,3	14	73,7
	Branca	4	18,2	2	10,5
	Sem informação	2	9,0	-	-
	Indígena	-	-	1	5,3
	Amarela	-	-	-	-
Cargo					
	Enfermeiro(a)	12	54,5	7	36,8
	Cirurgião(ã)-dentista	4	18,2	4	21,1
	Médico(a)	3	13,6	1	5,3
	Assistente social	2	9,1	1	5,3
	Técnico(a) de enfermagem	1	4,5	1	5,3
	Gerente	-	-	5	26,2

### Condições de Trabalho

Os efeitos nocivos à saúde dos trabalhadores decorrentes do trabalho precário apareceram destacados nas falas dos profissionais. A insuficiência de recursos humanos expõe os trabalhadores a longas e extenuantes jornadas de trabalho, situação agravada no cenário pandêmico, quando houve suspensão do gozo de férias e/ou concessão de períodos inadequados de descanso.

Não existe uma substituição de férias […] Não existe substituição de aposentadoria. Certo? Não existe substituição de licença-prêmio. […] a gente ficou dois anos sem poder fazer isso. Então, o déficit de RH é muito grande […] Acaba sobrecarregando as outras pessoas (UBS 21, sexo feminino, enfermeira).

Formas de contratação precarizadas da força de trabalho, como a pejotização, implicando a ausência de garantias trabalhistas a profissionais em relação inclusive ao direito à saúde, os quais estavam ali para promovê-la, de modo que a ausência no trabalho para submissão a exames diagnósticos ou cuidado com a saúde era penalizada financeiramente.

Aqui eu sou PJ [pessoa jurídica] […]. Eu tenho todas as obrigações de servidor público, mas não tenho as vantagens do servidor público. […] se eu ficar doente por Covid, eu consigo ser afastada e eles pagam por aquilo. Mas se eu ficar doente por qualquer outra coisa, […] eu perco os dias em que eu faltar. Quem cuida não é cuidado. […] agora dá suporte *pra* quem é funcionário público (UBS 17, sexo feminino, médica).

Destaca-se nos relatos dos trabalhadores a criação de um *locus* institucional específico de proteção da saúde dos trabalhadores durante o enfrentamento da Covid-19, contudo, de forma tardia e insuficiente.

Toda essa questão de colocar o profissional na linha de frente, assim, foi uma grande exposição […] acho que quase um ano depois […] Com o NAAT, isso facilitou bastante, porque você chega na hora marcada, vai e é avaliado (UBS 16, sexo feminino, enfermeira).

Vários funcionários estavam adoecendo emocionalmente, com um nível de desgaste muito alto [….]. Eles fizeram um grupo de trabalho na Secretaria para dar suporte emocional e trabalhar as equipes que se sentissem fragilizadas emocionalmente nessa rotina do Covid (UBS 19, sexo feminino, assistente social).

Sobre esse serviço (Núcleo de Apoio e Atendimento ao Trabalhador — NAAT) específico para os trabalhadores da saúde, instituído durante a pandemia para sua proteção, há convergência dos discursos no que se refere a sua importância na produção do cuidado. Os entrevistados destacaram ainda a importância da continuidade da oferta desse serviço após o período pandêmico, visando à permanência do cuidado da saúde dos profissionais de saúde.

Ainda bem que ele existiu, mas ainda precisava dele existir de forma mais forte […]. É uma coisa essencial, não dá *pra* acabar, vai ter que ser eterno isso aí (UBS 03, sexo feminino, médica).

Foi bastante importante na questão do acolhimento […], dessas orientações. Era o único local em que a gente tinha esse contato com a Gestão, porque o NAAT representava a Gestão *pra* gente, né? […] eu acho que foi muito importante existir. Tinha as estratégias de testagem dos profissionais, que eu acho que foi uma retaguarda importante que o Município criou (UBS 04, sexo feminino, enfermeira).

### Condições de Saúde

Durante a pandemia, os profissionais de saúde estiveram expostos frequentemente a trabalho exaustivo e associados a um contexto de forte tensão, necessitando da incorporação de saberes e habilidades (Quadro 2).

**Quadro 2:** Dimensões relacionadas às condições de saúde dos profissionais de saúde de um distrito sanitário de uma capital do Nordeste do Brasil, 2020–2022.

Dimensão	Subdimensão	Evidências relacionadas à saúde dos trabalhadores
Condições de saúde	Saúde mental	**2.1 — Processo de trabalho e suas repercussões na saúde dos profissionais** "Enfim, eu acho que o primeiro impacto foi essa questão de redimensionar as atividades, de reaprender o trabalho. De repreender, não. Aprender diante dessa nova realidade, que era uma realidade pandêmica. O segundo ponto, para as equipes de saúde, foi ter fôlego para dar conta do serviço sem perspectiva de término […]." (UBS 10, sexo feminino, assistente social) "[…] o que eu achei que foi muito difícil foi o estresse gerado nos profissionais de saúde, né? E pela insegurança, pela falta do conhecimento de como lidar com a situação, né? Pela sobrecarga de serviço. Houve muita sobrecarga de serviço […]." (UBS 9, sexo feminino, enfermeira) **2.2 — Desafios enfrentados pelos trabalhadores em saúde no contexto da pandemia** "Então, eu tinha medo de a gente adoecer e eles transmitirem, pegar por meus pais [….]." (UBS 2, sexo feminino, enfermeira) "Sem fazer atividade física, presa em casa, só sai *pra* trabalhar e volta […]. Então, acho que foi a parte mais difícil […]." (UBS 5, sexo feminino, enfermeira) "Na realidade, *pra* mim, foi um grande susto a Covid-19, a doença. Foi inesperado, foi assustador, desafiador, porque, além de ser linha de frente em tudo, a gente, além de estar enquanto profissional lidando com essa nova doença, a gente tinha todos os medos que as pessoas comuns têm […]." (UBS 1, sexo feminino, dentista) "Na minha vida, em particular, foi devastador, porque eu perdi minha mãe *pra* Covid. E foi na época que eu *tava* atendendo. […] a gente foi obrigado a atender sem nenhum suporte, sem nenhum treinamento, sem material básico *pra* atender […]." (UBS 21, sexo feminino, enfermeira) **2.3 — Cansaço físico e emocional da força de trabalho em saúde** "E uma outra coisa que está deixando, principalmente a equipe de enfermagem, muito cansada é a questão da vacinação, porque a mesma equipe de vacina é a mesma equipe que está atendendo aqui, é a mesma equipe que trabalha no final de semana. A gente aqui vacinou contra Covid por oito meses, de janeiro até agosto, então, a gente ficava estafada […]." (UBS 2, sexo feminino, enfermeira) "Eu acho que foi o mais desafiador de todos, porque as relações de trabalho ficaram extremamente estreitas, ariscas. Todo mundo se sentindo extremamente sobrecarregado, cansado, injustiçado […]." (UBS 16, sexo feminino, enfermeira) **2.4 — Repercussões na saúde mental** "A gente ia ter que fazer testagem. Então, foi desesperador mesmo… Sensação de medo, de pânico. Tiveram vários colegas com depressão. A gente teve colegas afastados por depressão, né? Eu tive pânico no primeiro momento, mas eu não cheguei a ter depressão, não cheguei a ter nenhum problema psíquico, não, mas eu fiquei muito desesperada… Todos tiveram aquele momento de pânico. E, assim, quando falam de heróis, não falam de heróis de quem está na unidade básica de saúde, não, né? As pessoas valorizam quem está dentro de hospital, mas tem que entender que a gente também é exposto […]." (UBS 12, sexo feminino, dentista) "A síndrome de *burnout* está por aí e tem necessariamente vários casos de colegas que estão adoecidos e afastados do serviço, né? Em decorrência da pandemia de uma forma geral e tudo que isso gera. Mas isso em si tem causado uma sobrecarga nos que ficam, e que estão adoecendo de outros problemas de saúde, né? E, assim, essa falta de reconhecimento dos profissionais que estão ali dando o sangue, né? É muito decepcionante! Porque os profissionais estão trabalhando com unhas, dentes e estão ali na linha de frente, linha de frente mesmo. Mas essa falta de reconhecimento… Então, a sobrecarga, é aos sábados, aos domingos, e você tem que trabalhar de qualquer jeito e tem que ver. E a gente não consegue, sabe? Aí a gente, tem uma hora que… Assim, realmente… A saúde mental, né? […]." (GF 02, UBS 18, sexo feminino, enfermeira)
	Implicações de gênero no processo saúde-doença	**2.5 — Influência dos marcadores sociais de gênero no adoecimento das trabalhadoras** "[…] eu não vou dar conta, porque eu estou trabalhando; você não tem noção do ritmo que eu estou trabalhando lá, sem folga, sem nada; como é que eu vou acompanhar as meninas nessa questão da tecnologia? […]." (UBS 16, sexo feminino, enfermeira) "Eu acho que, na minha experiência, eu tive que manter um equilíbrio muito grande… Com todo o processo dentro da sua casa, são filhos que precisavam ter alguém dentro de casa, porque estava tendo aula *online*; eu passei por isso, né? […]." (UBS 10, sexo feminino, enfermeira)
Condições de saúde	Covid longa	**2.6 — Covid longa** "Tem falta de ar, que eu tenho até hoje. Eu subi essa escada aqui, que são dez degraus. Eu subo no último, eu tenho que parar *pra* estabelecer minha respiração *pra* voltar. Fiquei com uma taquicardia grave, que outro dia eu estava aqui, eu estava com 180 batimentos cardíacos [por minuto]. E a perda de memória. Eu fui *pro* neuro e tudo mais, mas fiquei com algumas sequelas. Não existe um acompanhamento, como a neuro me disse. Eles não têm remédio *pra* lidar, não tem o tratamento *pra* fazer, porque até hoje a gente tem que aprender a conviver com ele, né? E aí, a gente que está na área de saúde, a gente consegue compreender isso […]." (UBS 21, sexo feminino, enfermeira) "Eu digo a você que, depois que se tem Covid, nunca mais você é a mesma pessoa. Eu digo isso, eu digo que meu cabelo caiu… Foi, meu cabelo caiu. Eu tenho um colega que disse que ficou esquecido. Então, é ampliar essa coisa da psicologia *pros* funcionários, até mesmo *pros* usuários […]. Eu conheço pessoas que dizem que ficaram com insônia […]." (UBS 18, sexo feminino, técnica de enfermagem) "Muitos dos nossos colegas estão com sequelas graves, que eu não sei nem se voltam mais *pra* trabalhar […]." (UBS 13, sexo feminino, assistente social)

UBS: unidade básica de saúde; GF: grupo focal.

Além das mudanças no cotidiano e no processo de trabalho, os profissionais da APS que atuaram na linha de frente no combate da Covid-19 tinham preocupações relacionadas ao medo de se infectar e infectar outras pessoas, levando-os à privação do convívio familiar, a mudanças constantes nos protocolos assistenciais, dado o desconhecimento da doença, e à falta de equipamentos de proteção individual. Esse cenário de extrema pressão foi responsável por intenso desgaste emocional dos trabalhadores e perdurou por grande parte do enfrentamento da pandemia mas, em especial, no período anterior ao da vacinação (Quadro 2, 2.2).

Com o avanço da pandemia, a força de trabalho em saúde foi submetida ao estresse crônico, e o desgaste provocado pelo prolongamento da carga horária repercutiu nas relações de trabalho e no bem-estar emocional, tornando os trabalhadores da APS ainda mais vulneráveis. A suspensão dos períodos de férias e folgas em boa parte do período da pandemia acentuou o cansaço e o adoecimento dos trabalhadores (Quadro 2, 2.3).

Observou-se que o medo, o pânico e suas repercussões fizeram parte da narrativa dos profissionais que atuaram na APS. As alterações de ordem psíquica apareceram de forma destacada, em especial a depressão e o sentimento de desvalorização. A pandemia tornou os ambientes assistenciais mais sobrecarregados, guardando relação recíproca com o adoecimento dos trabalhadores (Quadro 2, 2.4).

Observou-se desigualdade na manifestação do adoecimento dos trabalhadores e das trabalhadoras. Os relatos evidenciaram o acúmulo de responsabilidades das trabalhadoras também no âmbito domiciliar, tornando as sequelas da pandemia ainda mais contundentes entre as mulheres, destacadamente enquanto permaneceram as medidas de suspensão de atividades coletivas, como escolas, creches e outros equipamentos sociais (Quadro 2, 2.5).

Além de estresse físico e emocional, decorrentes do ambiente laboral em meio à pandemia, alguns trabalhadores que tiveram Covid-19 passaram a conviver com o prolongamento dos sintomas, apresentando um quadro de Covid de longa duração. As entrevistas destacaram sintomas como falta de ar, perda de memória e insônia, entre outras condições de saúde. Nesse contexto, alguns profissionais relataram dificuldade no desenvolvimento das suas atividades laborais (Quadro 2, 2.6).

## DISCUSSÃO

Os principais achados deste estudo demonstraram que o agravamento da precarização do trabalho em saúde e a insuficiência de medidas institucionais de proteção dos profissionais durante a emergência sanitária implicaram repercussões na sua saúde mental, em especial entre mulheres.

Não há dúvidas de que a pandemia tornou as fragilidades relacionadas a condições de trabalho e de saúde ainda mais evidentes. A precarização do trabalho possui as próprias dimensões, podendo ser entendida como reflexo da reestruturação produtiva^2^. O esgotamento derivado do prolongamento da jornada de trabalho, exponenciado pela desproteção social e por problemas emocionais, potencializou negativamente a saúde dos trabalhadores, gerando impactos que extrapolaram as fronteiras da faceta biológica do processo saúde-doença^9^.

Contratos de trabalho por meio de pessoa jurídica ou contratos temporários contribuíram para aumentar a vulnerabilidade dos profissionais, comprometendo sua remuneração após afastamentos por motivo de doença, inclusive a testagem para Covid-19^10^. De modo geral, a vinculação da força de trabalho em saúde com esses tipos de contratação vem acompanhada de um discurso de flexibilidade e liberdade, muitas vezes apreciadas pelos trabalhadores, embora, na prática, promovam sobrecarga de trabalho, transferência de riscos e custos aos próprios trabalhadores decorrentes da supressão de direitos trabalhistas^11^.

Esses vínculos frágeis e sem proteção social levam ao presenteísmo, que consiste no ato de o profissional comparecer ao trabalho com sinais e/ou sintomas de adoecimento, fenômeno que ocorre em vários locais e repercurte negativamente na saúde do trabalhador e nas instituições^12^. A ausência de proteção social e o receio de perder o emprego fazem com que indivíduos, inclusive médicos, como apontado no presente estudo, compareçam ao trabalho mesmo quando seu afastamento é necessário ou recomendado, como apontado na fala de uma das profissionais entrevistadas (Quadro 2)^13^.

Convém acrescentar ainda a repercussão na saúde derivada do vínculo precário, uma vez que a desproteção social pode implicar o comprometimento da assistência à saúde e da remuneração durante o afastamento do trabalho.

Outro ponto importante no enfrentamento da pandemia é a proteção fisica e emocional dos trabalhadores. Durante a crise sanitária, os profissionais da linha de frente conviveram com sobrecarga de emoções negativas em seus ambientes laborais, o que demandava cuidado psicológico desde o início do problema^14^. Medidas institucionais de proteção dos trabalhadores são imprescindíveis para superação de uma pandemia.

Em alinhamento com o contexto federal^15^, a Secretaria Municipal de Saúde (SMS) publicou um Plano de Contingência para os Trabalhadores em 2020, tendo como um dos principais objetivos preservar a força de trabalho da rede municipal, incluindo a saúde física e mental dos servidores. Foi criado então um serviço, percebido pelos trabalhadores, como espaço de oferta de testagem, apoio psicológico e psiquiátrico durante a crise sanitária, que funcionou entre maio de 2020 e novembro de 2021, quando foi descontinuado^16^.

Em dezembro de 2023, o município publicou nota técnica com orientações gerais sobre medidas de prevenção e controle da Covid-19, não contemplando a proteção dos profissionais de saúde nas medidas de prevenção e controle da doença. Nesse documento, esse serviço de atenção exclusiva aos trabalhadores da SMS aparecia como oferta de testagem aos profissionais, demonstrando retrocesso institucional no cuidado com os trabalhadores.

A falta de compromisso dos gestores com as intervenções propostas constitui uma dificuldade de continuidade das ações de proteção dos trabalhadores^17^. Evidencia que, apesar do arcabouço jurídico disponível para execução das atividades previstas na Política Nacional de Saúde do Trabalhador e da Trabalhadora^18^, a prática de proteção dos profissionais é permeada por dificuldades.

O adoecimento dos profissionais de saúde pode reduzir os recursos humanos disponíveis para o enfrentamento da pandemia e comprometer a resposta dos serviços de saúde. O novo contexto laboral decorrente da pandemia impôs a necessidade de aprendizagem de protocolos assistenciais, dado o desconhecimento da doença. Os indivíduos que formavam a força de trabalho em saúde conviveram com o medo, a angústia e a sobrecarga de trabalho, entre outros fatores que podem ser considerados gatilhos para desencadeamento ou agravamento de doenças mentais^19,20^. Proteger a saúde física e mental dos trabalhadores da saúde é de fundamental importância para manutenção dos serviços e preservação da vida humana, em especial em contextos de crise sanitária.

Os profissionais de saúde conviviam também com a escassez de equipamentos de proteção individuais, isolamento social e carga horária extensiva de trabalho^21^.

Ademais, destacaram-se nas narrativas a sobrecarga de trabalho durante grande parte da crise sanitária. A ampliação do horário de trabalho reduziu a possibilidade da manutenção de um estilo de vida saudável, ampliando o risco de adoecimento^22^. A Organização Internacional do Trabalho^23^ destaca que os indivíduos que trabalham 48 horas por semana, presencial ou virtualmente, sofrem consequências negativas em curto e longo prazos.

O aumento no nível de estresse no ambiente laboral repercute em três principais problemas: esgotamento mental, transtornos de ansiedade e depressão^21^. A prevalência de sintomas de transtornos mentais, como ansiedade e depressão, em profissionais de saúde da APS, durante a pandemia, foi em torno de 43,2%, e a sobrecarga de trabalho é um dos elementos associados à presença de transtornos^24^.

Artigo de revisão sobre a saúde mental dos trabalhadores da linha de frente mostrou prevalências de sintomas de ansiedade variando de 44,6 a 62%, com cerca de 30% dos profissionais apresentando nível moderado ou grave de ansiedade, e 50% apresentando sintomas de depressão^25^.

Nesse contexto, a Organização Mundial da Saúde^26^ indicou aumento global na prevalência de ansiedade e depressão de 25% no primeiro ano da pandemia. O atendimento às medidas de distanciamento físico promoveu interrupções nos serviços de saúde mental, levando a lacunas no tratamento ofertado à população, sendo essencial ampliar os serviços de saúde mental e psicossocial, reconhecendo-os como componentes da cobertura universal da saúde em todo o mundo.

O estresse crônico proveniente do trabalho trouxe para alguns trabalhadores o desenvolvimento da síndrome de *burnout*, ou síndrome do esgotamento profissional. De modo geral, o impacto do adoecimento psíquico de profissionais de saúde traz consequências sociais, pessoais e institucionais, repercutindo no bem-estar das pessoas atendidas^27^.

Observa-se, portanto, que a saúde mental deve ser considerada como um dos elementos centrais nas políticas públicas para a saúde do trabalhador no Sistema Único de Saúde, tendo em vista o caráter essencial do serviço que esses profissionais prestam na atenção à população.

Ainda considerando as condições de saúde dos trabalhadores, fica evidente a influência da questão de gênero no adoecimento das trabalhadoras.

Os achados deste estudo corroboram a literatura revisada^22,28,29^ sobre a participação das mulheres na área da saúde: elas são maioria. No contexto da crise sanitária, acúmulo de vínculos trabalhistas, baixos salários, falta de compartilhamento das tarefas domésticas, entre outros elementos, contribuíram para aumentar o risco de infecção e repercussões na saúde física e mental das trabalhadoras^28^.

Levando em cosideração os diversos tipos de violência que estão expostas cotidianamente, as mulheres possuem três vezes mais possibilidade de apresentar transtornos mentais menores em relação aos homens^30^. Isto posto, a análise dos impactos da pandemia na saúde dos trabalhadores por meio do recorte de gênero expõe iniquidades e vulnerabilidades que permeiam as práticas laborais e a vida em sociedade.

Destaca-se ainda a identificação de trabalhadores com o prolongamento dos sintomas de Covid-19. No Brasil, o Ministério da Saúde optou pelo termo "condições pós-Covid" para denominar as alterações de saúde que permanecem ou se iniciam quatro semanas ou mais após infecção por Covid-19, não podendo ser enquadradas em outro diagnóstico^31^. Considerando a importância dessa condição no contexto do trabalho, a Agência Europeia para Segurança e Saúde no Trabalho elaborou guias para orientar gestores e empregadores para o retorno seguro e saudável dos profissionais com sintomas contínuos ao ambiente laboral, incluindo a manutenção do contato com o trabalhador durante o afastamento e a preparação para o seu retorno, considerando estratégias como regresso faseado (ou gradual) e ajustes nas funções e nos horários^32^.

Essa condição também tem implicações no desenvolvimento de novas doenças crônicas e, por conseguinte, os serviços de saúde devem estar preparados para lidar com essas novas demandas^33^, assim como as empresas e os serviços nos quais esses profissionais estão lotados.

Como limites desse estudo, destaca-se que os profissionais entrevistados pertenciam a um único DS e que a expansão da amostra poderia gerar reflexões mais robustas sobre as dimensões destacadas. Contudo, as evidências do potencial comprometimento da saúde dos trabalhadores infectados pela Covid-19 indicam a necessidade de acompanhamento desses profissionais e o estabelecimento de ações de coordenação do cuidado no contexto pós-Covid.

Analisando-se as condições de saúde e trabalho dos profissionais da APS, observou-se que o contexto singular da pandemia amplificou o adoecimento e abriu espaço para novos estressores. Vale ressaltar que a precarização do trabalho e, em especial, do trabalho em saúde, é resultado de processo histórico e não restrito ao Brasil. Compreender as implicações do avanço das políticas neoliberais e seus impactos na saúde dos trabalhadores e seu processo de trabalho são essenciais para o aprimoramento das políticas públicas e da qualidade da assistência prestada. Os resultados desta pesquisa colaboram para o entendimento dos elementos que atuam, de forma entrelaçada, na saúde do trabalhador.

Apesar de os problemas descritos não serem novos, não há dúvida de que a pandemia agravou as fragilidades. No centro da crise sanitária, permaneceram os trabalhadores de saúde, vivenciando no seu cotidiano problemas que já os permeavam antes mesmo da eclosão da pandemia.

## Disponibilidade de Dados:

Os dados da pesquisa não inclusos no artigo não estão disponíveis.
